# Sternal closure with sandwiched three-piece bioresorbable mesh reduces postoperative hemorrhage: a retrospective study

**DOI:** 10.1186/s13019-023-02460-6

**Published:** 2023-11-27

**Authors:** Yasutaka Yokoyama, Taira Yamamoto, Tetsuma Oyama, Jiyoung Lee, Yoichiro Machida, Daisuke Endo, Yuichiro Sato, Shizuyuki Dohi, Atsushi Amano, Minoru Tabata

**Affiliations:** 1 Department of Cardiovascular Surgery, Juntendo University School of Medicine, 2-1-1, Hongo, Bunkyo-ku, Tokyo, 113-8421 Japan; 2 Department of Cardiovascular Surgery, Toda chuo General Hospital, 1-19-3, Honcho, Toda, Saitama 335-0023 Japan

**Keywords:** Median sternotomy, Bioresorbable mesh plate, Postoperative sternal displacement, Postoperative Hemorrhage

## Abstract

**Background:**

Median sternotomy is the most performed procedure in cardiac surgery; however, sternal displacement and bleeding remains a problem. This study aimed to investigate whether sternal reconstruction using a sandwiched three-piece bioresorbable mesh plate can prevent postoperative sternal displacement and bleeding more than a bioresorbable pin.

**Methods:**

Patients (n = 218) who underwent median sternotomy were classified according to whether a sandwiched three-piece bioresorbable mesh plate and wire cerclage (group M, n = 109) or a bioresorbable pin and wire cerclage (group P, n = 109) were used during sternal reconstruction. The causes of postoperative sternal displacement and bleeding with computed tomography data were analyzed and compared between the groups.

**Results:**

The preoperative patient characteristics did not significantly differ between the groups. However, the evaluation of sternal and substernal hematoma on postoperative day 5 using computed tomography showed sternal displacement in 4 (4%) and 22 (20%) patients, and substernal hematoma in 17 (16%) and 41 (38%) patients in groups M and P, respectively; this difference was significant. Furthermore, the amount of bleeding at 6 h postoperatively was lower in group M than in group P (235 ± 147 vs. 284 ± 175 mL, p = 0.0275). Chest reopening, intubation time, and length of intensive care unit and hospital stays did not differ between the groups. The evaluation of substernal hematoma based on computed tomography yielded a significantly lower for group M than for group P, revealing that the mesh plate was an independent predictor of substernal hematoma prevention.

**Conclusion:**

Sternal fixation with a three-piece bioresorbable mesh plate could prevent postoperative sternal displacement, bleeding, and substernal hematoma more than sternal fixation with a pin.

## Background

Median sternotomy is the most common surgical procedure in cardiac surgery. The incidence of complications, such as sternal dehiscence and infections, associated with this procedure ranges from 0.3 to 8% [1, 2]. Furthermore, sternal wound complications increase the mortality rate from 10 to 40% [3].

**Fig. 1:**
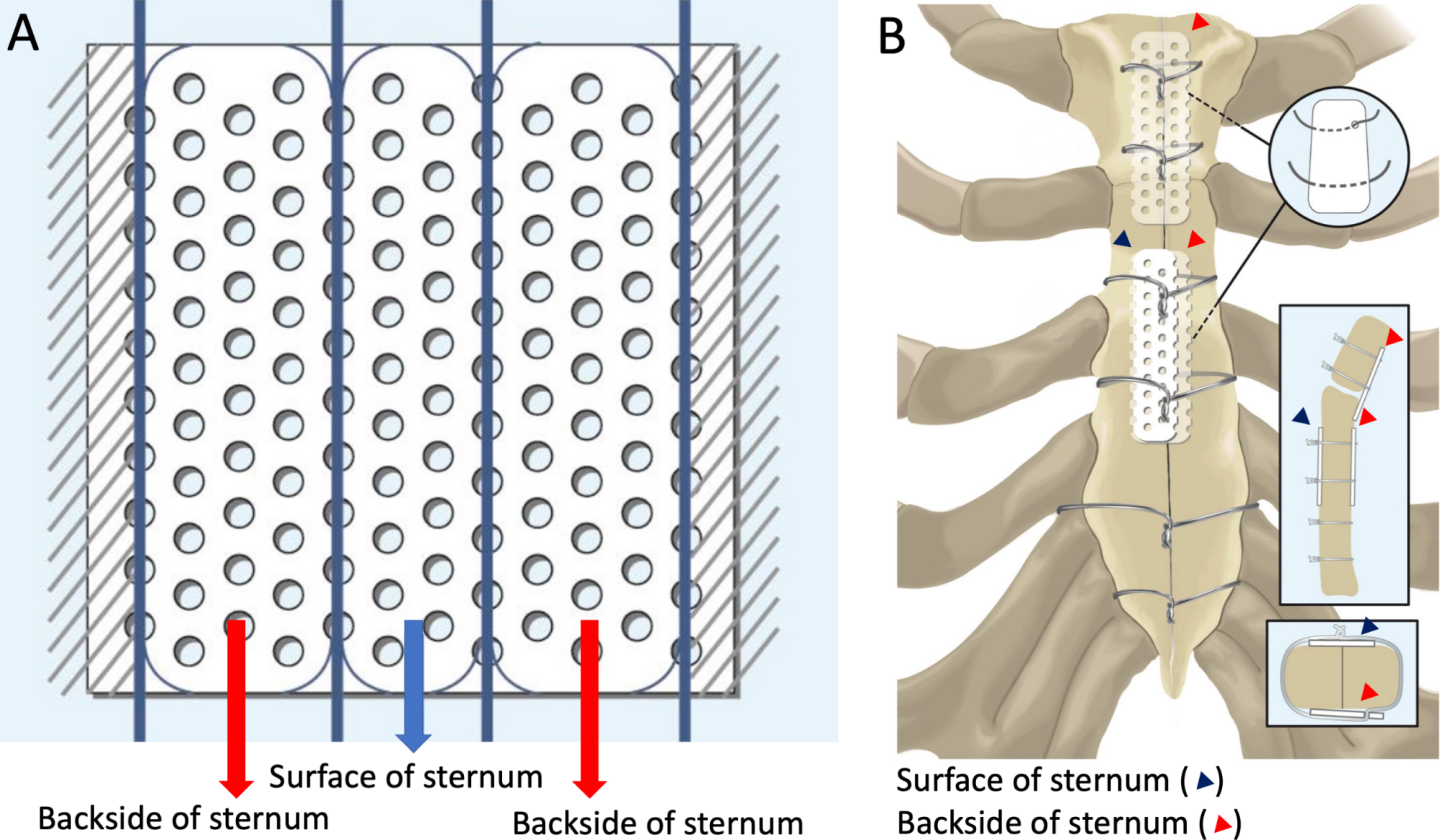
Schema of sternal closure with mesh plate. **A)** The mesh plate is cut into three pieces, and the four corners of each plate are cut using scissors. **B)** Chest closure: the sternum is sandwiched between three mesh plates; two on the posterior surface and one on the anterior surface

Approximately 6–25% of cases of thoracic sternal infection are associated with a reopened chest due to postoperative hemorrhage, indicating a causal relationship between hemorrhage and infection [4]. Bleeding from the sternum is caused by sternal displacement; therefore, preventing sternal displacement is one of the most crucial goals in cardiac surgery. Stainless steel wiring is the standard method for sternal closure after median sternotomy. Recently, Super FIXSORB MX40 mesh plates (Teijin Medical Technologies CO. Ltd., Osaka, Japan), which are made of poly-L-lactide, have been applied in addition to conventional stainless-steel wiring. The mesh plate is resorbed after approximately 6 years through osteoconduction [5] and has already been used for fixing rib fractures and flail chest repair [6, 7]. Furthermore, postoperative respiratory conditions are more stable when a mesh plate is used for median sternotomy after congenital cardiac surgery and coronary artery bypass grafting [8, 9]. Therefore, we devised a method to close the sternum by external fixation using a three-piece mesh plate (Fig. 1).

Fixation was found to be stronger with this technique, and it was inferred that prevention of sternal displacement would reduce the incidence of postoperative bleeding. In the present study, we hypothesized that chest closure with the mesh method would reduce the incidence of postoperative sternal hemorrhage by preventing postoperative sternal displacement and compared conventional chest closure using pins to that using mesh plates.

## Methods

### Study population

This retrospective study included 249 consecutive patients who underwent cardiac surgery at Toda Chuo General Hospital between April 2017 and December 2019. The following patients were excluded: 2 patients who underwent minimally invasive cardiac surgery, 1 with chest closure using SternaLock Blu (Biomet Microfixation CO. Ltd., Jacksonville, FL), 26 with chest closure using Fixsorb wave (Teijin Medical Technologies CO. Ltd., Osaka, Japan), and 2 who could not be withdrawn from cardiopulmonary bypass. Ultimately, the study included 218 patients.

For basic sternal fixation, conventional wire cerclage was used. Between July 2018 and December 2019, we used a three-piece mesh plate for full sternotomy (group M, n = 109). For comparison, group P (n = 109) included patients who underwent chest closure using a pin between April 2017 and February 2019. We selected patients based on the expectation that external fixation with mesh would be effective for group M due to the presence of sparse sternal marrow and internal fixation with sternal pins would be effective for group P. Data on the postoperative course, bleeding, infection, and computed tomography (CT) were compared between the groups. This study was approved by the Institutional Review Board of Toda Chuo General Hospital (approval number: 0517; received on November 29, 2021).

### Preoperative management

Off-pump coronary artery bypass grafting was performed with oral aspirin, and surgery using a cardiopulmonary bypass was performed 7, 5, 5, and 2 days after discontinuation of aspirin, clopidogrel, warfarin and novel oral anticoagulant, respectively. This preoperative management was routine practice for patients taking anticoagulant medication at our institution. It was not specifically discontinued for the purpose of this study.

### Surgical procedure

**Fig. 2:**
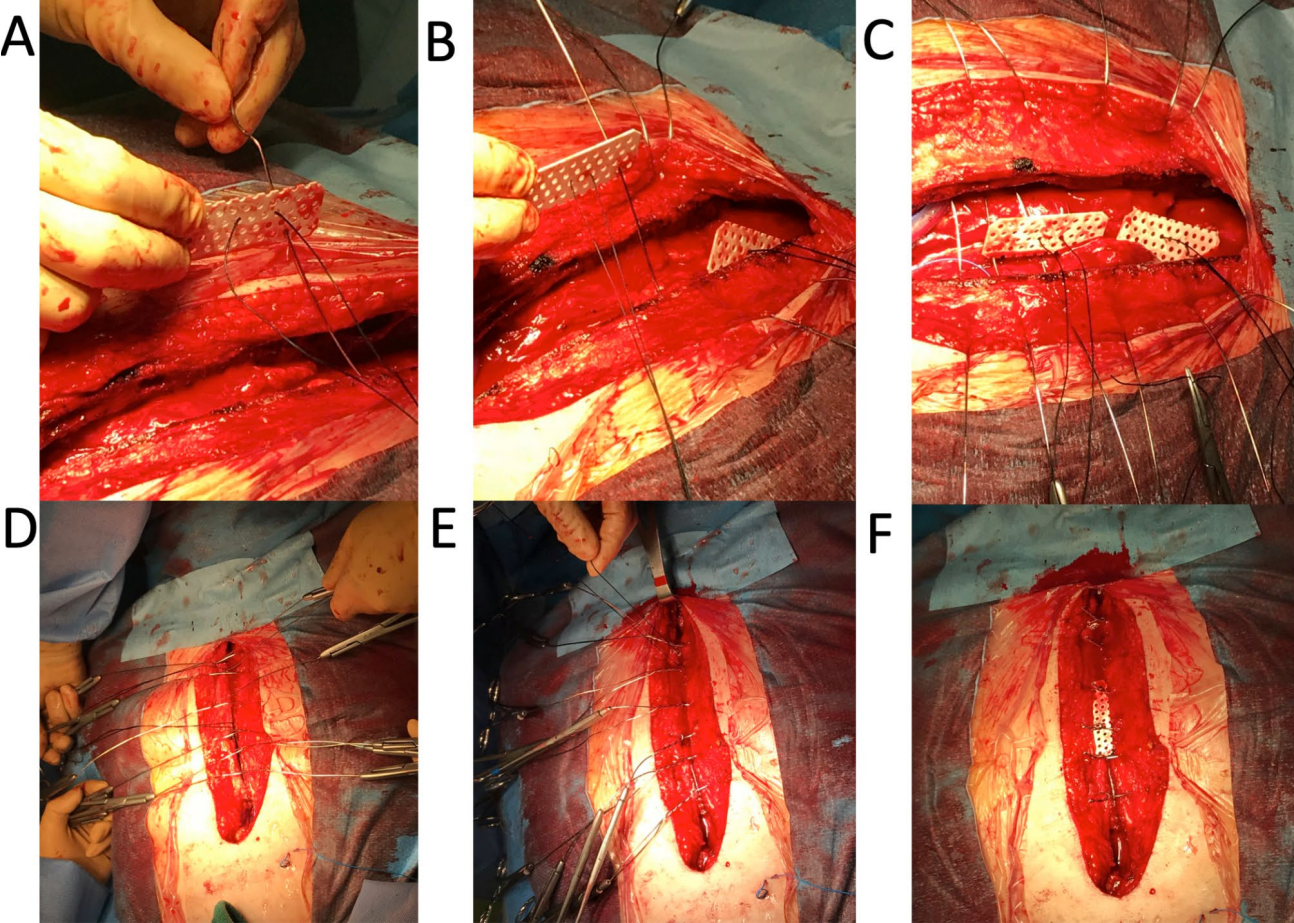
How to perform sternal closure with sandwiched three-piece mesh plate. **A)** The first mesh plate is threaded through the holes on the plate with the first manubrium wire. **B)** The second mesh plate is threaded through the holes with the third parasternal wire. **C)** The sternum is closed with six wires; two transsternal wires at the manubrium and four parasternal wires at the sternal body. **D)** When the sternum is closed, two mesh plates are implanted on the posterior side of the sternum. **E)** When closing the wire, the silk is towed so that the mesh plate fits on the posterior side of the sternum. **F)** The chest is closed using three mesh plates with the sternum sandwiched in between, with two mesh plates located posteriorly and another anteriorly

All patients underwent sternal closure with conventional wire cerclage without bone wax that was performed by a single surgeon. Six simple single wires were used; two transsternal and four parasternal wires were placed at the manubrium and sternal body, respectively. The mesh plate and all corners of each plate were cut with scissors (Fig. 1A). The schema of the mesh plate implantation method is shown in Fig. 1B. The mesh plate was fixed by passing two transsternal and parasternal wires (first and third) through the holes on the mesh plate. Subsequently, each wire was tied, and two mesh plates were implanted on the posterior surface of the sternum. The sternum was closed using three mesh plates; two on the posterior surface and one on the anterior surface. Figure 2 shows the procedure for sternal closure with a three-piece mesh plate. From deep to superficial, the pectoral fascia was closed with 0 Vicryl sutures in a continuous pattern. The subcutaneous tissue was closed with 2 − 0 Vicryl sutures in a continuous pattern after placement of a 10-Fr subcutaneous drain, and the skin was closed with 5 − 0 polydioxanone clear sutures in a continuous pattern. In group P, the above procedure was also performed except that instead of a mesh, one pin each was inserted into the sternal manubrium and body. All patients received prophylactic antibiotic treatment with cefazolin (dosage based on individual patient weight) for 48 h postoperatively.

### Postoperative management

Once admitted to the postoperative intensive care unit (ICU), activated clotting time was measured hourly and maintained at < 120 s by administering protamine as needed. Postoperative blood glucose levels were monitored hourly and maintained at < 180 mg/dL by continuous intravenous insulin administration. Drains were placed in the pericardial and thoracic cavities as well as retrosternally. The effluent volume was measured hourly with milking of the drainage as needed. Sedation was discontinued when the drainage rate was < 30 mL/h, and the patient was extubated from the ventilator when he was fully conscious and oriented. The criterion for leaving the ICU was having a hemodynamically stable condition and being able to walk 50 m. All patients underwent chest CT on postoperative day 5 to assess the sternum displacement and check for substernal hematoma.

### Definitions and endpoints

**Fig. 3:**
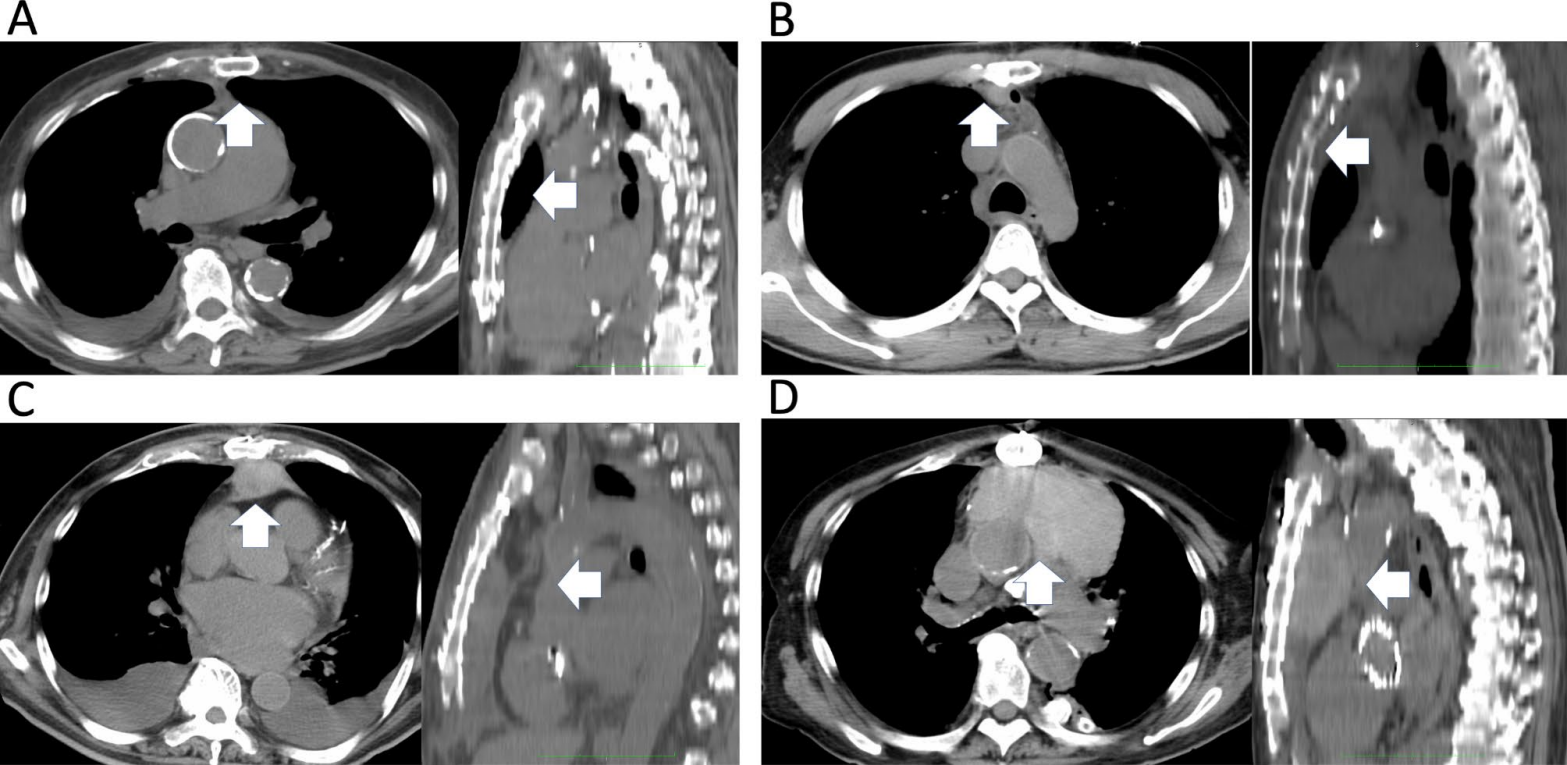
Grade of substernal hematoma. **A)** Substernal hematoma is not present on CT. **B)** Slight substernal hematoma is seen on CT. **C)** Substernal hematoma equal to or larger than the size of the sternum, up to twice the size. **D)** Substernal hematoma larger than twice the size of the sternum. CT, computed tomography

The primary endpoint was the postoperative sternum displacement. The secondary endpoints included the amount of bleeding, length of ICU and hospital stays, rate of deep sternal wound infection, degree of substernal hematoma (Fig. 3), sternal healing, and dehiscence using CT data on postoperative day five. After cardiac surgery, because of the incision of the pericardium, blood from the sternum may flow into the pericardial cavity and be aspirated. Therefore, we used the total drainage volume, including bleeding from the sternum, as an indicator to more accurately assess the amount of bleeding from the sternum. To evaluate sternal healing, we adopted methods from previous studies [10, 11]. Deep sternal wound infection was defined according to the following criteria: (1) bacteria could be isolated from cultures of mediastinal tissue or fluid; (2) evidence of mediastinitis was observed during surgery; and (3) presence of chest pain, sternal instability, or fever (> 38℃) with either purulent mediastinal discharge or bacterial isolation from a blood culture of drainage fluid originating from the mediastinal area [12].

### Statistical analyses

Continuous and normally distributed data are presented as means ± standard deviations, while categorical data are expressed as percentages. All analyses were performed using JMP Pro version 15 (SAS Institute, Cary, NC, USA). Statistical significance was set at p < 0.05. Between-group differences were evaluated using the Chi-square test for categorical variables and the t-test or Mann–Whitney U test for continuous variables. We compared the baseline characteristics, operative data, and postoperative complications of groups P and M using the Pearson 2 test for categorical variables, the t-test for continuous variables, and the Mann–Whitney U test for continuous skewed data. We evaluated independent predictors for preventing substernal hematomas using logistic regression analysis. We categorized CT-detected substernal hematomas as ‘hematoma present’ if they were Grade 2 or higher, and as ‘hematoma absent’ if they were Grade 1.

## Results

The preoperative patient characteristics are listed in Table 1. No significant differences were observed between the two groups, including in terms of platelets and the coagulation system of PT, APTT, and PT-INR. Preoperative drug prescriptions, including aspirin, clopidogrel, warfarin, or novel oral anticoagulants, were not significantly different between the groups. Additionally, both groups did not significantly differ in the preoperative diagnoses, including ischemic heart disease, aortic stenosis, aortic regurgitation, mitral stenosis, mitral regurgitation, aortic aneurysm, and aortic dissection. The SPPB (Short Physical Performance Battery) is an assessment that includes three components: the Balance test (4 points), Gait speed test (4 points), and Chair stand test (4 points), with a total score of 12 points. A score < 9 points indicates frailty. No significant difference in frailty was noted between the two groups.

**Table 1:** Patient characteristics

	M (*n* = 109)	P (*n* = 109)	*p*-value
Age (years)			
Under 60	16 (15%)	22 (20%)	0.4125
60–69	21 (19%)	26 (24%)	
70–79	43 (39%)	40 (37%)	
Over 80	29 (27%)	21 (19%)	
Sex (men), *n* (%)	67 (61.5%)	75 (68.8%)	0.2555
Body mass index (kg/m^2^)	23.1 ± 3.6	22.9 ± 3.7	0.6833
Hypertension, *n* (%)	80 (73.4%)	71 (65.1)	0.1865
Dyslipidemia, *n* (%)	53 (48.6%)	46 (42.2%)	0.3410
DM, *n* (%)	29 (26.6%)	20 (18.3%)	0.1442
Smoking history, *n* (%)	59 (54.1%)	59 (54.1%)	1.0000
HD, *n* (%)	2 (1.8%)	6 (5.5%)	0.1496
History of stroke, *n* (%)	9 (8.3%)	10 (9.2%)	0.8102
Orthopedic disease, *n* (%)	22 (20.2%)	16 (14.7%)	0.2841
Hb (g/dL)	12.7 ± 2.0	13.0 ± 1.9	0.1888
Platelet (x10^3^/µL)	20.7 ± 6.5	20.3 ± 5.6	0.6192
Alb (g/dL)	3.9 ± 0.5	4.0 ± 0.5	0.2320
eGFR (mL/min/1.73 m^2^)	57.9 ± 19.5	60.7 ± 22.6	0.3376
BNP (pg/mL)	238.4 ± 553.1	246.7 ± 491.4	0.9136
PT (sec)	13.1 ± 2.2	12.9 ± 2.5	0.5298
PT-INR	1.22 ± 0.30	1.30 ± 0.46	0.1137
APTT (sec)	34.8 ± 9.0	34.1 ± 6.1	0.4794
HbA1c (%)	6.0 ± 1.0	5.9 ± 0.7	0.4071
Ejection fraction (%)	63.2 ± 12.7	62.3 ± 14.4	0.6304
**Preoperative drugs**			
Aspirin	23 (21.1%)	27 (24.7%)	0.5191
Clopidogrel	10 (9.2%)	11 (10.1%)	0.8184
Warfarin	3 (2.8%)	6 (5.5%)	0.3071
NOAC	17 (15.6%)	13 (11.9%)	0.4316
**Diagnosis**			
Ischemic	21 (19%)	25 (23%)	0.8254
Aortic Stenosis	21 (19%)	17 (15%)	
Aortic Regurgitation	11 (10%)	12 (11%)	
Mitral Stenosis	1 (1%)	3 (3%)	
Mitral Regurgitation	23 (21%)	22 (20%)	
Aneurysm	19 (18%)	14 (13%)	
Dissection	10 (9%)	10 (9%)	
Others	3 (3%)	6 (6%)	
Pre-operative SPPB	11.3 ± 1.4	11.3 ± 1.3	0.8575
Euro Score (%)	6.7 ± 9.7	5.7 ± 6.8	0.3904

DM, diabetes mellitus; HD, hemodialysis; Hb, hemoglobin; alb, albumin; eGFR, estimated glomerular filtration rate; BNP, brain natriuretic peptide; HbA1c, hemoglobin A1c; NOAC, novel oral anticoagulant; SPPB, short physical performance battery

Operative data, such as the procedure (rates of coronary artery bypass grafting, valve, aorta, concomitant, and congenital surgeries), rate of emergency, amount of bleeding, volume of blood transfused, and operative, perfusion, cross-clamp, and upper and lower circulatory arrest times were not significantly different between the groups. These details are presented in Table 2.

**Table 2:** Operative results

	M (*n* = 109)	P (*n* = 109)	*p*-value
CABG, *n* (%)	18 (17)	21 (19)	0.4625
Valve, *n* (%)	44 (40)	41 (38)	
Aorta, *n* (%)	16 (15)	21 (19)	
Concomitant, *n* (%)	26 (24)	22 (20)	
Congenital, *n* (%)	0	2 (2)	
Others, *n* (%)	5 (5)	2 (2)	
Emergency, *n* (%)	19 (17%)	24 (22%)	0.4967
Amount of bleeding (mL)	744 (279–1165)	526 (233–1046)	0.4362
Amount transfused (mL)	2370 (1851–2945)	2440(1975–3198)	0.1965
Blood transfusion, *n* (%)	105 (96%)	108 (99%)	0.1747
Amount of blood transfused (mL)	800 (415–1292)	755 (400–1432)	0.1136
Operation time (min)	300 ± 96	298 ± 107	0.6980
Perfusion time (min)	151 ± 58	146 ± 48	0.3541
Cross-clamp time (min)	113 ± 48	110 ± 44	0.1122
Upper circulatory arrest time (min)	7.7 ± 4.3	8.6 ± 4.4	0.4854
Lower circulatory arrest time (min)	47.7 ± 17.2	49.4 ± 19.2	0.7389

CABG, coronary artery bypass grafting

The postoperative course is summarized in Table 3. The displacement of sternum (4 [4%] vs. 22 [20%], P = 0.0002) significantly differed between groups M and P. The total volume of blood loss from all drains (235 ± 147 vs. 284 ± 175 mL, p = 0.0275) and the number of patients with > 400 mL blood loss (8 [7%] vs. 22 [20%], p = 0.0059) at 6 h postoperatively significantly differed between groups M and P, respectively. No significant difference was found between the groups regarding the rates of chest reopening for bleeding, thoracocentesis, sternal infection, intubation time, and lengths of ICU and hospital stays.

**Table 3:** Postoperative course

	M (*n* = 109)	P (*n* = 109)	*p*-value
Displacement of sternum	4 (4%)	22 (20%)	0.0002
6 h total amount of bleeding (mL)	235 ± 147	284 ± 175	0.0275
Over 400 mL/6 h bleeding, *n* (%)	8 (7%)	22 (20%)	0.0059
Reopen chest for bleeding, *n* (%)	0	2 (1.8%)	0.1554
Thoracocentesis, *n* (%)	1 (0.9%)	0	0.3162
Intubation time (min)	412 (145–7,935)	465 (195–46,080)	0.2692
Over 48 h intubation time, *n* (%)	7 (6%)	6 (6%)	0.7749
ICU stay (days)	2.0 ± 2.6	2.8 ± 5.0	0.1575
Over 2 days ICU stay, *n* (%)	32 (29%)	41 (38%)	0.1965
First day of sitting (days)	1.5 ± 1.9	1.2 ± 0.7	0.1346
First day of standing (days)	2.0 ± 4.9	1.3 ± 0.9	0.1567
First day of walking (days)	1.9 ± 3.7	1.8 ± 1.5	0.8046
Able to walk 50 m (days)	1.9 ± 4.9	2.2 ± 2.6	0.6095
Able to walk 100 m (days)	2.8 ± 1.5	3.2 ± 2.7	0.2008
Able to walk 200 m (days)	5.2 ± 5.0	4.6 ± 3.3	0.3533
Able to walk 300 m (days)	5.6 ± 5.2	5.1 ± 2.4	0.3970
Hospital stay (days)	14.3 ± 9.7	13.8 ± 12.0	0.7467
Sternal infection, *n* (%)	1 (0.9%)	0	0.3162

ICU, intensive care unit

The postoperative CT findings are listed in Table 4. No significant differences were observed in the sternal displacement in the coronal section or sternal gap, dehiscence, and cutting. However, the sternal displacement in axial images and substernal hematoma were significantly lower in patients from group M than in those from group P. The substernal hematoma (17 [16%] vs. 41 [38%], P = 0.0002) significantly differed between groups M and P. The four-grade evaluation of substernal hematoma on CT yielded Grade 1, 2, 3, and 4 values of 145 (76%) vs. 15 (58%), 19 (10%) vs. 8 (31%), 23 (12%) vs. 2 (8%), and 5 (3%) vs. 1 (4%) in displacement and those without, respectively (p = 0.0229). Furthermore, the four-grade evaluation of substernal hematoma on CT yielded Grade 1, 2, 3, and 4 values of 92 (85%) vs. 68 (62%), 8 (7%) vs. 19 (17%), 8 (7%) vs. 21 (19%), and 1 (1%) vs. 1 (1%) in groups M and P, respectively (p = 0.003). Logistic regression analysis using the substernal hematoma on CT showed that the use of a mesh plate was an independent predictor of substernal hematoma prevention (Table 5).

**Table 4:** Postoperative computed tomography findings

	M (*n* = 109)	P (*n* = 109)	*p*-value
Sternum step-off of axial section	4 (4%)	22 (20%)	0.0002
Sternum step-off of coronal section	0	0	1
Gap of sternum	3 (2%)	2 (2%)	0.6510
Dehiscence of sternum	0	0	1
Cutting of sternum	0	0	1
Substernal hematoma	17 (16%)	41 (38%)	0.0002
Postoperative hematoma grade			
Grade 1	92 (85%)	68 (62%)	0.0030
Grade 2	8 (7%)	19 (17%)	
Grade 3	8 (7%)	21 (19%)	
Grade 4	1 (1%)	1 (1%)	

**Table 5:** Multivariable predictors for substernal hematoma on postoperative computed tomography

	Univariate		Multivariate	
	OR (95% CI)	*p*-value	OR (95% CI)	*p*-value
Age (years)	1.00 (0.98–1.03)	0.7518	0.98 (0.95–1.02)	0.3193
Sex (men) Yes = 1	0.91 (0.67–1.25)	0.5673		
HD Yes = 1	1.04 (0.49–2.76)	0.9166	0.98 (0.35–2.32)	0.9623
Orthopedic disease Yes = 1	1.10 (0.74–1.69)	0.6541	0.86 (0.52–1.35)	0.5242
Platelet count	0.98 (0.94–1.03)	0.4883		
Anticoagulant medication Yes = 1	1.15 (0.76–1.69)	0.4819	1.02 (0.56–1.77)	0.9540
Euro Score (%)	1.01 (0.97–1.05)	0.7068		
Emergency Yes = 1	1.12 (0.77–1.60)	0.5485		
Intraoperative bleeding (mL)	1.00 (1.00–1.00)	0.7672		
Operation time (min)	1.00 (1.00–1.00)	0.9589		
Perfusion time (min)	1.00 (1.00–1.01)	0.1518		
Cross-clamp time (min)	1.01 (1.00–1.01)	0.0995	1.01 (1.00–1.02)	0.2095
Mesh Yes = 1	0.55 (0.40–0.76)	0.0003	0.58 (0.40–0.82)	0.0028

OR, odds ratio; CI, confidence interval; HD, hemodialysis

**Fig. 4:**
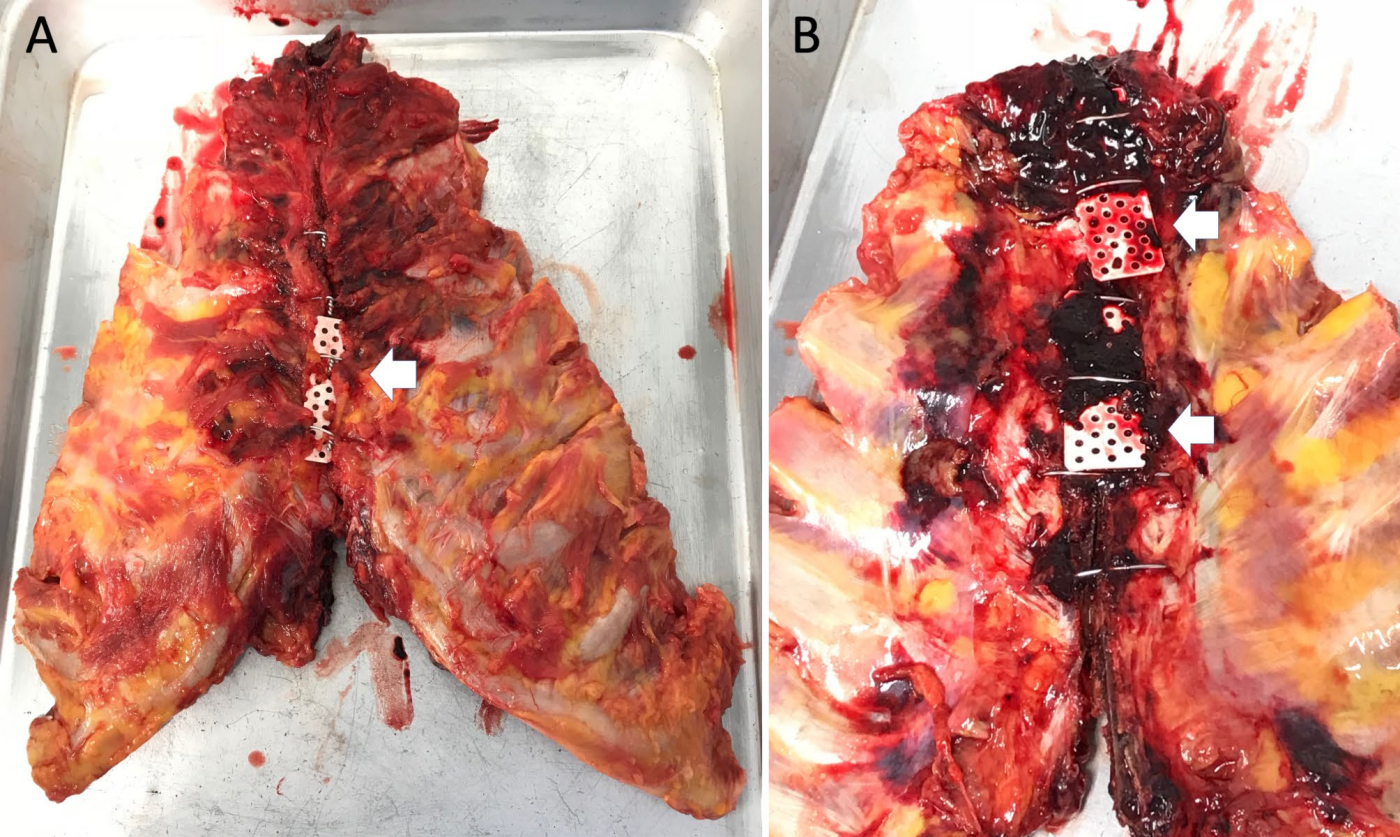
Sternum in an autopsied case. Sternal surface in autopsied case. **A)** The sternum is not displaced because it was sandwiched between three plates. **B)** Posterior surface of the sternum in the autopsied case. The posterior plates acted as a hemostatic despite the presence of a hematoma

Of the two patients who could not be withdrawn from cardiopulmonary bypass, one underwent autopsy in whom the mesh plate on the posterior sternal surface was tightly bound to the back of the sternum wherein hematoma occurred. In this case, despite the presence of percutaneous cardiopulmonary support, the patient had good hemostasis and minimal blood loss, with a total blood loss volume of 430 mL after 6 h. The autopsy confirmed that the sternum was not displaced because it was sandwiched between three plates, and that the posterior plate acted as a hemostatic despite the presence of a hematoma (Fig. 4).

## Discussion

Sternal closure using three pieces of a bioresorbable mesh plate that sandwiches sternal halves could reduce the incidence of sternum displacement, postoperative bleeding, and substernal hematoma when compared to sternal closure with a bioresorbable pin. In our study, sternal closure using mesh plates reduced the incidence of bleeding in the postoperative period, which may prevent postoperative infection and improve wound healing.

There have been eight reports of sternal closure using bioresorbable plates. Tanaka et al. reported that postoperative respiratory and hemodynamic conditions were more stable with Super FIXSORB MX40 mesh plates for median sternotomy after congenital cardiac surgery compared to unused. [8]. Additionally, Tamura et al. reported that fixation with Super FIXSORB MX40 mesh plates resulted in increased sternal stability, decreased postoperative pain, and improved postoperative respiratory function [9]. As shown in Fig. 1, we devised this method assuming that sandwiching the sternum between the plates would prevent sternal displacement and postoperative bleeding by tightly aligning the sternal bone marrow. Axial CT evaluation on postoperative day 5 showed a significantly less sternal step-off and less postoperative substernal hematoma formation in group M than in group P (Table 4), which was similar to the study by Watanuki et al. who reported less anteroposterior sternal displacement [13]. In our multivariate analysis of postoperative CT data for substernal hematoma, the mesh plate was an independent predictor of substernal hematoma prevention.

Our closure method does not require preoperative bone marrow width measurement; however, our method requires a wire to be placed between the ribs of the sternal body to implant the mesh retrosternally. As a result, depending on its size, the wire penetrating the sternum may be less than the width of the mesh and could interfere with sternal closure. In contrast, preoperative bone marrow sizing is required prior to pinning. Additionally, the bone marrow width does not correlate with sternal body size, and reports have emphasized the importance of preoperative bone marrow size measurement. The study concluded that the pin size in the bone marrow should be measured by preoperative CT [14]. Since the mesh acts as an external fixator, preoperative measurements are unnecessary, unlike the case with pins, which are internal fixators. The mesh is expected to provide better fixation than pins for patients with a sparse sternal marrow, such as in older women with osteoporosis. Two mesh pieces are placed posteriorly because there is less muscle and connective tissue on the posterior surface of the sternum than on the anterior surface, and the tight fit and crimping may prevent bleeding. Meanwhile, we used a narrower mesh on the anterior surface so as not to interfere with the closure of the muscles and connective tissue. Imanishi et al. treated a sternal fracture with a mesh plate sandwiched between the front and back of the sternum for fixation [15].

**Fig. 5:**
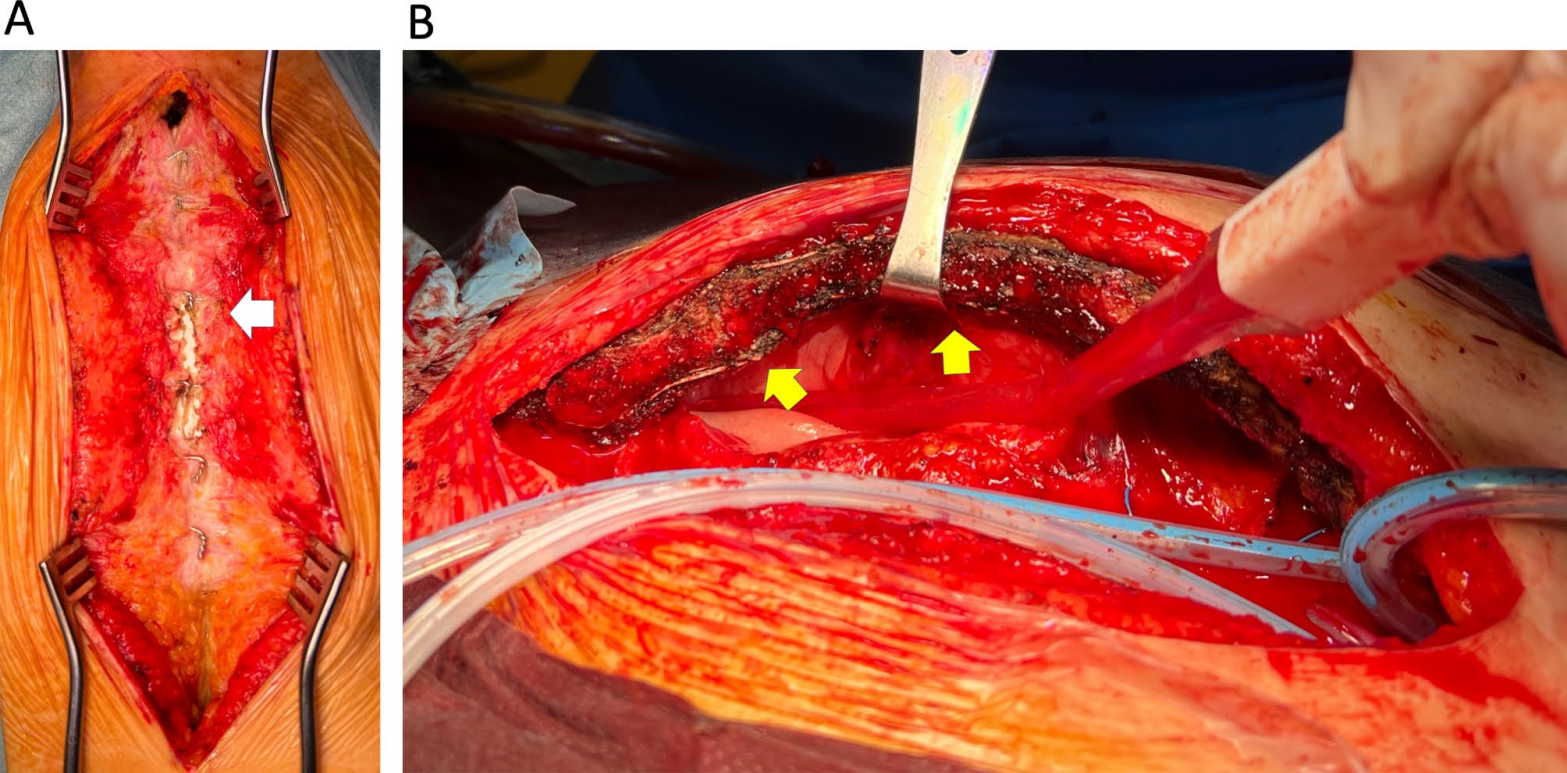
Intraoperative photo of the reopened chest 2 years postoperatively. **A)** Sternal surface of a reopened chest. (white arrow). **B)** Posterior aspect of a reopened chest. (yellow arrow)

At the time of reoperation, which was 2 years after the initial surgery, the chest was opened with a sternal saw (Fig. 5). The mesh plate was not an obstacle during the reoperation as it could be cut in a manner similar to the Super FIXSORB MX40, which is a bioresorbable mesh plate composed of unsintered or uncalcined hydroxyapatite and poly-l-lactic acid. Although this sheet is resorbed in the body after approximately 6 years [16, 17], no reports have indicated that Super FIXSORB MX40 mesh plate increases the risk of inflammation or infections. In the present study, the incidence of mediastinitis was similar between both groups, suggesting that there are no disadvantages to using a mesh plate.

This study had some limitations. First, our study was retrospective, since a sample size was not determined before conducting the research, the results may not indicate a statistically sufficient number of cases. In future studies, an adequate sample size should be evaluated before commencing the research for a more detailed examination of the results obtained in this study. Second, this was a single-center study; therefore, it was limited by the relatively small number of included patients. Owing to the small number of cases, the statistical power of the analysis may not be sufficient. Third, unmeasured confounding factors may have influenced the results. Therefore, further prospective studies with a large cohort are required.

## Conclusion

Sternal closure with a three-piece bioresorbable plate sandwiching the sternum is a safe method that could reduce the incidence sternum displacement, postoperative bleeding, and substernal hematoma formation when compared to sternal closure using a bioresorbable pin.

## Data Availability

The datasets used and/or analyzed during the current study are available from the corresponding author on reasonable request.

## List of abbreviations

CT: computed tomography
ICU: intensive care unit
SPPB: Short Physical Performance Battery
DM: diabetes mellitus
HD: hemodialysis
Hb: hemoglobin
Alb: albumin
eGFR: estimated glomerular filtration rate
BNP: brain natriuretic peptide
HbA1c: hemoglobin A1c
NOAC: novel oral anticoagulant
CABG: coronary artery bypass grafting
